# Effect of LHCGR Gene Polymorphism (rs2293275) on LH Supplementation Protocol Outcomes in Second IVF Cycles: A Retrospective Study

**DOI:** 10.3389/fendo.2021.628169

**Published:** 2021-05-11

**Authors:** Ramaraju GA, Ravikrishna Cheemakurthi, Madan Kalagara, Kavitha Prathigudupu, Kavitha Lakshmi Balabomma, Pranati Mahapatro, Sivanarayana Thota, Aruna Lakshmi Kommaraju, Sanni Prasada Rao Muvvala

**Affiliations:** ^1^ Center for Assisted Reproduction, Krishna IVF Clinic, Visakhapatnam, India; ^2^ Department of Biotechnology, Institute of Science, Gitam (Deemed to be) University, Visakhapatnam, India

**Keywords:** LHCGR gene, LHCGR 312, polymorphism, IVF, ART, pharmacogenomics

## Abstract

Infertility is a major concern for couples wanting to have progeny. Despite recent advances in the field of IVF, success rates still need improvement. Understanding the patient’s variability and addressing it with personalized interventions may improve the success rate of fertilization and live births. This study examined the impact of a personalized pharmacogenomic approach on LH supplementation on the pregnancy and live birth rate outcomes in comparison with the traditional approaches. 193 patients undergoing a second IVF cycle in Krishna IVF Clinic received LH supplementation either as per the conventional methods or based on N312S (rs2293275) LHCGR gene polymorphism. Results showed a significant increase in pregnancy rate (P-value: 0.049) and a trend showing improvement in live birth rates (P-value: 0.082) when r-hLH supplementation protocol was decided as per the genotypes A/A, A/G, and G/G of the N312S variant in the respective patients. This stimulation regimen helped in providing optimum levels of r-hLH supplementation to patients with impaired hormone-receptor interacting activity, to achieve higher success in pregnancy and live birth rates.

## Introduction

Infertility is a growing health problem worldwide due to stress, increased exposure to pollutants, delayed marriages, and various socio-economic reasons (1). In cases where the natural reproductive process is suboptimal and fails to achieve a successful pregnancy, assisted reproductive technology (ART) may help to improve the chances of successful pregnancy and live birth. In ART, agonistic or antagonistic protocols (2, 3) are used to interrupt the natural process of ovulation, followed by stimulation using either human menopausal gonadotropins or recombinant human Follicle Stimulating Hormone(r-hFSH) along with recombinant human Luteinizing Hormone (r-hLH).

In the case of controlled ovarian stimulation (COS), it was understood that FSH alone was adequate to achieve better stimulation of follicles. But later studies showed that supplementation of LH along with FSH had additional effects in some groups. However, the specific groups are not identified and the optimum dosages are not established.

Initially, women were classified as poor and/or hypo-responders for ovarian stimulation but the definition of these groups was not clear. Ferraretti and Gianaroli on behalf of The European Society of Human Reproduction and Embryology, established the Bologna criteria to identify poor responders (4). Later Alviggi et al. on behalf of “The Patient-Oriented Strategies Encompassing Individualized Oocyte Number group” (POSEIDON) updated the criteria for low ovarian reserves and poor/hypo-responders (5–9).

LH supplementation plays a crucial role in COS. In the case of the hypo-responders with adequate ovarian reserves, the ovarian response to the stimulation protocol improved when LH supplementation was added to the FSH doses (3, 10–12). LH assists growth, improves development and triggers ovulation of mature follicles. LH is also critical for the differentiation of mature follicle to the corpus luteum, and maintain normal luteal function to ensure proper gonadal functioning (13). In our earlier study, in 2012 we reported better pregnancy rates when we replaced the use of r-hFSH plus hMG with r-hFSH plus r-hLH combination (14). Interestingly, we observed variable responses among the women receiving r-hLH supplementation.

Luteinizing hormone (LH) acts through luteinizing hormone/human chorionic gonadotrophin receptor (LH/CGR). LHCGR is expressed on theca cells and subsequently developed on granulosa cells (15). Polymorphisms in the genes of FSH, LH, and the coding regions of their respective receptors have been studied extensively (16). The LHCGR gene is located on chromosome 2, with 11 exons at cytogenetic band 2P16.3. Over 520 SNPs have been identified in the LHCGR gene. The SNP (rs2293275) occurs at position 935 of exon-10, resulting in the replacement of asparagine with serine (N312S) in the LHCGR protein.

FSH consumption in COS protocol is studied and reviewed in relation to FSHR, LHβ, and LHCGR gene polymorphisms (17). No significant difference was observed in FSH consumption, the mean number of oocytes or mature oocytes retrieved among the patients with different LHCGR (rs2293275) genotypes. However, a significant difference was observed in the live birth rates among the different LHCGR genotypes (18, 19). Few studies demonstrated that the presence of asparagine at 312 position in LHCGR may render the receptor more sensitive (20, 21). The N312S polymorphism is also linked to polycystic ovary syndrome (PCOS), where the heterozygous and homozygous asparagine group has 2-fold and 3-fold increased risk of having PCOS (22). The role of r-hLH supplementation in relation to LHCGR polymorphisms was not studied extensively when compared to FSH. In our study in 2018 on LHCGR N312S polymorphism, we found a strong association between LHCGR polymorphism and the requirement of r-hLH in COS protocols and pregnancy outcome. Evidence indicates that r-hLH and r-hFSH co-administration improves follicle to oocyte yield and pregnancy rates in hypo-responders rather than in poor responders, establishing an individualized pharmacogenomics tool based on LHCGR polymorphism. The present study is a retrospective analysis of a group of patients with an unsuccessful first *in vitro* fertilization cycle and undergone a second cycle at Krishna IVF clinic. In the second cycle, the stimulation was optimized either based on the patient’s LHCGR N312S genotype or P.C.Wong criteria. The primary objective of the present study is to fix the need and optimum dosage of LH supplementation based on LHCGR polymorphism (N312S) and the secondary objective is to know the effect of adopting pharmacogenomic approach in LH supplementation on take-home baby success rates.

## Materials and Methods

### Study Design

A study was designed on patients (N = 281) who underwent a second IVF cycle from January 2008 to December 2018 in Krishna IVF and the data were extracted from the database. Out of 281 cases, 193 women with unsuccessful first IVF cycle, that subsequently underwent a second cycle at Krishna IVF Clinic, were included in the analysis. Exclusion criteria include patients stimulated with hMG/bravelli (n=66), those subjected to antagonist protocol (n=17), those involved in donor cycles (n=3), and those failed to follow-up (n=2). Inclusion criteria include all patients who underwent the second cycle following agonist protocol with stimulation performed using recombinant r-hFSH and r-hLH. Patients were divided into two groups: Group-I consisted of 78 patients receiving LH supplementation as P. C. Wong criteria (LH dosage of 75 IU/day supplemented from day-6 onwards) while Group-II patients were provided with LH supplementation based on their SNP profile in LHCGR gene polymorphism (A/A, A/G, and G/G alleles). The same long luteal gonadotropin-releasing hormone (GnRH) agonist down regulation was initiated in the patients of both groups and comparable supplementation with FSH was used. Patients were given the choice to choose the conventional protocol or the protocol based on SNP profiling. The strength of the study includes personalized pharmacogenomic approach in reproductive medicine. Limitations of the study are small sample size and retrospective analysis. Large prospective study in different ethnic groups will have better objectivity. Informed consent was obtained prior to the study from the participants, and the study was conducted in accordance with the declaration of Helsinki. The study was approved by the local Institutional Ethics Committee for research on human volunteers, Krishna IVF Clinic, Visakhapatnam, India (Approval reference number: KIVF/LEC/09/16-17).

### Stimulation Protocol

A long luteal phase GnRH agonist protocol was used for ovarian stimulation. Ovarian suppression achieved with a single dose of decapeptide depot injection containing Triptorelin 3.75 mg (Ferring Pharmaceuticals, Saint-Prex, Switzerland) administered intramuscularly between the 18^th^ to 24^th^ day of the menstrual cycle. After 14 days, downregulation was confirmed by serum estradiol levels of < 50 pg/mL and endometrium thickness of < 5 mm. Then the follicular recruitment and development are ensured by ovarian stimulation by the administration of a daily subcutaneous injection of r-hFSH (Gonal-f, Merck Serono SA, Switzerland). The dose of r-hFSH was fixed in the range of 150 IU to 300 IU per day based on age, BMI, and Antral Follicle Count (AFC). The same dosage continued during the entire course of stimulation.

In the second cycle for the Group-I patients, the LH supplementation was given based on P. C. Wang criteria for all the A/A, A/G, and G/G genotype patients. Post-ovarian stimulation from day-6 onwards all the Group-I patients are supplemented with 75IU/day of LH.

**Figure 1:**
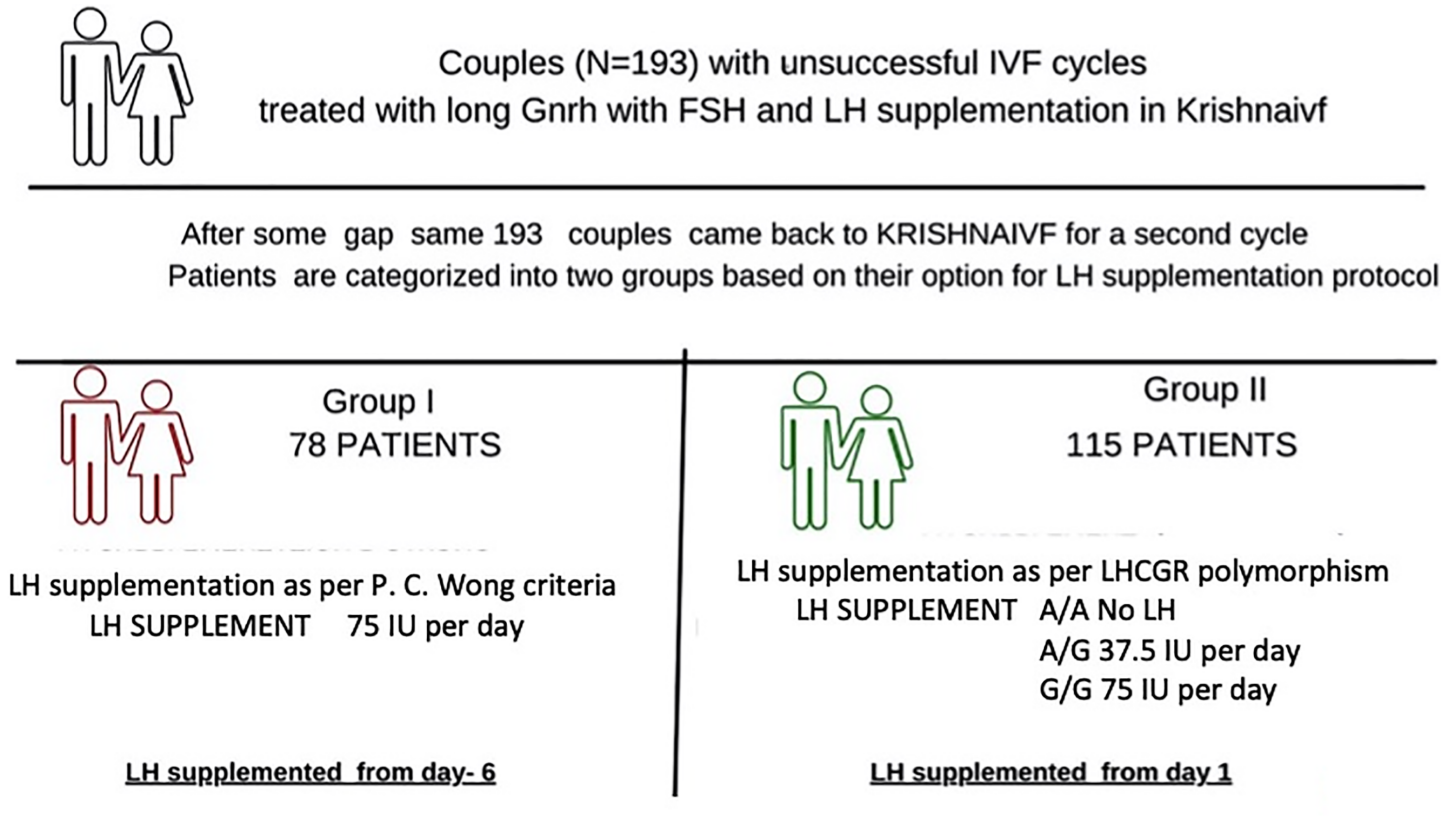
Flow chart for stimulation protocol followed.

For the Group-II patients in the second IVF cycle, LH supplementation was provided based on the LHCGR (N312S) polymorphism concept. Here the A/A group received no LH supplementation while the A/G group received 37.5 IU and the G/G group 75.0 IU. For these patients, starting from day one of ovarian stimulation, the respective LH supplementation dosage was maintained throughout the stimulation for each polymorphic group in addition to the common r-hFSH protocol (**Figure 1**).

### Genotyping

The genetic assessment of polymorphisms in the LHCGR gene for A/A, A/G, and G/G variants was performed as previously described (19).

### Egg Collection, Fertilization, and Embryo Transfer

The progress of oocyte development was monitored during the treatment through vaginal ultrasound scans and by monitoring estradiol levels. Human chorionic gonadotrophin(hCG) was administered when the follicle size reached ≥ 18mm as assessed by ultrasound. Eggs were retrieved after 34-38 hours following hCG administration through ultrasound guidance under conscious sedation. Collected oocytes were graded and fertilized with individual sperm using the intra-cytoplasmic sperm injection (ICSI) method followed by embryo culture as described earlier (19). The fertilization of oocytes was confirmed by the formation of pronuclei after 18 hours of ICSI. After fertilization embryos were progressed in the culture medium for the next 48 hours. On day 4, embryo grading was performed on a morphological basis using Veeck’s criteria (23). Blastomeres with equal size and no fragmentation in the embryo were considered to be grade-I, and blastomeres with equal/unequal sizes and minimal to complete fragmentation were considered as Grade-II embryos. The hCG levels in plasma were checked after 14 days of embryo transfer to confirm the pregnancy. Further confirmation is done by ultrasound by looking for the formation of the gestational sac. Live births were registered for all the women.

The evaluation of LHCGR gene polymorphisms, the collection of patient demographic data, stimulation protocols, assessment of the quality of oocytes and the embryos, and decision on the number of embryos transferred were done in similar conditions in all the cases. Similarly, the same techniques and procedures under identical conditions were used for hormone analysis, egg retrieval, intra-cytoplasmic sperm injection, culture media preparation for growing embryos, and embryo transfer. The chemicals and drugs used are of the same grade and procured from the same source.

### Statistical Analysis

Statistical analysis was performed using R Studio Desktop version 3.6.2 (free open source edition). Descriptive statistics (percentages [%], mean, and standard deviation [SD]) were calculated and stratified by treatment Groups (I and II). The difference in the mean values for all numerical variables between the 1^st^ cycle and two groups of 2^nd^cycle (age, BMI, marital life, AFC) were performed using one-way ANOVA. The mean values for all numerical variables between Group-I and Group-II of the second cycle were performed using an unpaired two-sample independent t-test. For r-hLH supplementation, the difference in mean values was calculated using an independent t-test among genotype groups (A/A, A/G, G/G) and between the Group-I and Group-II of the second cycle, to show the dose variation among genotypes. The difference in clinical pregnancy rates and live birth rates were calculated using the chi-square test. P values <0.05 were considered to be significant. AFC was measured based on the criteria proposed by Broekmans et al. (24). If the largest follicle size is >10mm in diameter then the other follicles with ≤ 10mm size were identified leaving the largest follicle and if the largest follicle size is ≤10mm in diameter all the follicles are counted. Clinical pregnancy and the live birth rates were calculated based on ICMART definition. The number of clinical pregnancies expressed per aspiration cycles, and the number for live birth deliveries expressed per aspiration cycles. The calculated effect size considering the minimum number of samples (n=78), in each group is 0.45, which according to Cohen’s et al. (25) classification is a medium effect for 0.05 level of significance, and the calculated power is 0.81 for dependent variable r-hLH dose using t-test. The power for chi-square analysis shows 0.79 with a calculated effect size of 0.314 for a minimum sample size of n=78.

## Results

Basal characteristics, such as the age of male and female, marital life, female body mass index, and AFC are summarized in **Table 1** during the 1^st^ and 2^nd^ cycles. The mean age of the couples and marital life as expected was significantly different during the second cycle compared to the first cycle. There were no significant differences in the BMI of the patients and AFC during the first and second cycles.

**Table 1:** Basal characteristics of the first and second cycle.

Variable	1^st^ Cycle (n = 193)	2^nd^ Cycle (Group-I) (n = 78)	2^nd^ Cycle (Group-II) (n = 115)	P-Value
Female Age (in years)	29.42 ± 4.04	30.28 ± 3.74	31.48 ± 4.14	<0.001
Male Age (in years)	35.44 ± 4.33	35.76 ± 4.19	37.83 ± 4.53	<0.001
Marital Life (months)	77.92 ± 36.42	91.17 ± 38.05	100.03 ± 42.97	<0.001
Body Mass Index (BMI)	26.89 ± 3.86	26.24 ± 4.78	26.43 ± 4.80	0.24(NS)
Antral follicle count (AFC)	12.02 ± 3.35	13.09 ± 3.59	12.39 ± 3.03	0.054(NS)

NS, Not Significant.

### Hormonal Supplementation

Total FSH or FSH/day doses for Groups-I and II are comparable and hence the role of FSH is supposed to average out while comparing the results of the second cycle for the two groups. No significant difference was observed in the total FSH or FSH/day between the two groups.

In the second cycle, the mean total r-hLH was significantly different between group-I and II among genotypes A/A (416.97 ± 232.66 IU, 0.00 ± 0.00 IU, P-value: <0.001) and G/G (351.14 ± 142.60IU, 738.72 ± 144.81IU, P-value: <0.001). However, no significant difference was observed in the A/G (432.56 ± 321.66 IU, 404.31 ± 99.25IU, P-value: 0.58) genotype (**Table 2**).

**Table 2:** Unpaired two sample independent t-test between 2^nd^ cycle (Group-I) and 2^nd^ cycle (Group-II).

Variable	2^nd^ cycle (Group-I) N = 78	2^nd^ cycle (Group-II) N = 115	P-Value
Female Age (in years)	30.28 ± 3.74	31.48 ± 4.14	0.042
Male Age (in years)	35.76 ± 4.19	37.83 ± 4.53	0.001
Body mass index (BMI)	26.24 ± 4.78	26.43 ± 4.80	NS
Marital Life (months)	91.17 ± 38.05	100.03 ± 42.97	NS
Antral Follicle count (AFC)	13.09 ± 3.62	12.39 ± 3.03	NS
Total r-hFSH dose, IU	2271.49 ± 634.01	2408.22 ± 701.30	NS
r-hFSH IU/day	208.45 ± 44.63	222.40 ± 71.33	NS
Total r-hLH dose, IU AA AG GG	407.44 ± 266.03 416.97 ± 232.66 432.56 ± 321.66 351.14 ± 142.60	533.08 ± 274.17 0.00 ± 0.00 404.31 ± 99.25 738.72 ± 144.81	0.001 <0.0001 0.58 <0.0001
r-hLH IU/day (mean)	57.38 ± 25.11	52.59 ± 25.66	NS
Treatment days (mean)	10.85 ± 1.37	10.88 ± 1.25	NS
LH Days (mean)	6.45 ± 3.62	9.05 ± 3.67	<0.0001
Fertilization (%)	84.53	86.19	NS
Number of oocytes	13.83 ± 5.02	13.36 ± 4.82	NS
Mature oocytes	9.88 ± 5.12	10.45 ± 4.62	NS
Embryo quality (G-I)	1.50 ± 1.63	1.37 ± 1.39	NS
Embryo quality (G-II)	3.33 ± 1.77	3.62 ± 2.03	NS
Day 4 Embryos	4.79 ± 2.05	5.05 ± 2.45	NS
Embryos transferred	3.74 ± 1.13	3.67 ± 1.14	NS
Clinical pregnancy rate (N) (%)-positives	26(33.3%)	56(48.7)	0.049
Live birth rate (N) (%)	19(24.4)	45(39.1)	0.082

NS, Not Significant.

### Comparison of Groups-I and II of the Second Cycle

**Figure 2:**
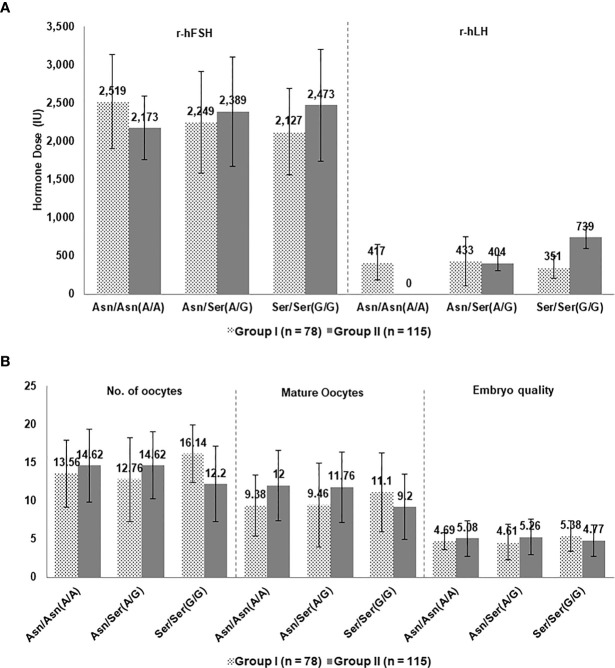
**(A)** Hormone supplementation and **(B)** Embryology parameters for patients in Groups I and II in the second cycle based on LHCGR (Asn312Ser) variations.

**Figure 3:**
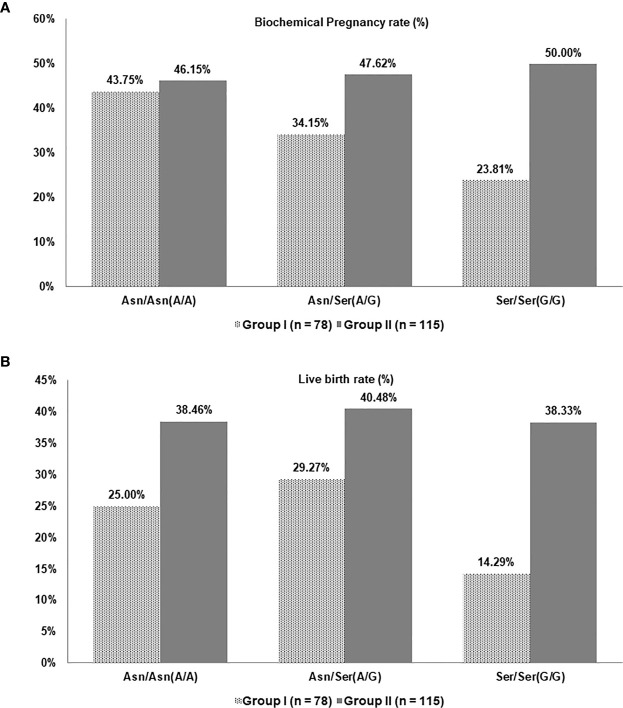
**(A)** Biochemical pregnancy rate and **(B)** Live birth rate for patients in Groups-I and II in the second cycle based on LHCGR (Asn312Ser) variations.

Since the A/A, A/G and G/G data are available for both the groups at the time of undergoing the second IVF cycle, data for Groups-I and II were compared based on each genotype. FSH and LH supplementation protocols were compared for the three alleles of A/A, A/G, and G/G in the second cycle and data regarding egg and embryo quality and pregnancy were assessed (**Figures 2**, **3**). As seen in **Figure 2A**, the amount of FSH supplemented to patients with different genotypes and between Groups-I and II was similar. On the other hand, the amount of LH supplemented to patients with A/A and G/G genotypes were significantly different between Groups-I and II (**Figure 2A**). The number of oocytes, mature oocytes, and embryo quality was slightly better in A/A and A/G genotypes of Group-II while the values for the G/G genotype were better in Group-I (**Figure 2B**).

In the second cycle, in patients that underwent conventional LH supplementation with the P. C. Wong protocol (Group I), the average biochemical pregnancy rates observed were 43.75%, 34.15%, and 23.81% in patients with genotype A/A, A/G, and G/G, respectively (**Figure 3A**). The biochemical pregnancy rate does not show a significant difference for A/A genotype in patients that underwent the pharmacogenomic based protocol (Group II) but showed improved clinical pregnancy rates of 47.62% and 50.00%, for genotypes A/G and G/G respectively.

Regarding live birth rates, patients undergoing conventional LH supplementation with the P. C. Wong criteria (Group-I) had 25.00%, 29.27%, and 14.29% success rate in the second IVF cycle for genotypes A/A, A/G, and G/G, respectively (**Figure 3B**). However, an improved live birth rate was observed when genotype-based LH supplementation was used (Group-II), with 38.46%, 40.48%, and 38.33% rates were observed for the three genotypes A/A, A/G, and G/G, respectively.

The second IVF cycles for Groups-I and II were performed under similar conditions and protocols except for the LH supplementation. Thus the higher success rates in the second group suggest measured LH supplementation stimulation protocol may improve success rates in patients with A/G and G/G variants in the LHCGR gene.

### Age and Marital Life

At the time of the second cycle, patients in Group-II had a mean age of 31.5 years with mean marital life of 8.3 years (about 100), had a pregnancy rate of 49.69%, and a live birth rate of 39.13%. These rates were higher than the historical data from http://ivfpredict.com, with predicted pregnancy rates of 24.3% and 23.1% for the age groups of 29 and 32 years respectively, and with a marital life of six years. This difference suggests that personalized protocols based on specific genotypes may yield comparatively higher clinical pregnancy and live birth rates.

Results from patients with A/A genotype suggest that LH supplementation was not required to increase pregnancy and live birth rates. In fact, the supplementation of r-hLH in AA genotype patients has decreased live birth by 13.46%. On the contrary, an additional LH dosage of 37.5 IU in the A/G group and 75 IU in the G/G group, improved pregnancy and live birth rates.

## Discussion

In the current study, patients with failed first IVF cycle returning to the clinic for the second cycle were classified into two groups according to the stimulation protocol. One group was treated with a pharmacogenomic approach and the other with traditional stimulation based on P.C. Wong criteria. The treatment protocol was identical for both groups except for LH stimulation.

Group-II patients undergoing LH supplementation through the pharmacogenomic approach had higher pregnancy and live birth rates than the Group-I patients treated based on the P.C. Wong criteria. Therefore, optimized LH supplementation protocols might contribute to a higher clinical pregnancy rate in patients with specific genotypes, rather than the number and morphological characteristics of the embryos retrieved. These favorable conditions might be responsible for the small differences in the maturation of the oocyte, usually not noticeable under a microscope with some changes taking place after the hCG administration or at a later developmental stage after embryo transfer.

This observation is in line with that of Lindgren et al. (18), where they observed no significant differences in the number of follicles or oocytes or embryo quality between those carrying LHCGR allele Asn312 (A/A) and Ser312 (G/G) in their study population but the pregnancy rates differed markedly. Moreover, a recent report that the number of oocytes retrieved in IVF cycles has no relation to birth weight z-scores and the ovarian response is not related to adverse perinatal and obstetric outcomes (26), further supports our observation.

A careful examination of 6-8 years of unsuccessful marital life and the multiple IVF cycles undergone by the patients do not meet the favourable prognosis criteria and hence, on average 3 embryos are transferred based on ASRM guidelines (27).

The key changes in the protocols are described here for both groups:

1. During the second IVF cycle for the patients in Group-I, the long luteal phase GnRH agonist protocol with LH supplementation was followed based on P.C.Wong criteria for ongoing Low/Hypo Responders concept.
2. In the second cycle for the Group-II patients, LH supplementation was given based on the LHCGR 312 polymorphism concept. For the A/A genotype, no LH supplementation was given, for the A/G genotype, 37.5 LH supplementation was used and 75.0 IU of LH supplementation was used for patients with G/G genotype. The rest of the protocol remained the same as that of the previous ART cycle.
3. The FSH supplementation remained comparable for the two groups in both cycles.

Both FSH and LH stimulate their ovarian target cells by activating their highly specific FSH and LH receptors. These receptors belong to the G protein-coupled receptor family and signal through the classical 3′, 5′- cyclic adenosine monophosphate (cAMP)/protein kinase A pathway (28). These two hormones are essential for normal follicular development and adequate oocyte maturation (29).

Angers et al. (30), studied the granulosa cells and proposed that the FSH and LH receptors form homo- and heterodimers so that stimulation by one of the hormones could be mediated in part through the other hormone’s receptor. Thus, they proposed that the LHCGR genotype could influence the response to FSH stimulation. Lindgren et al. (18) attempted to elucidate the mechanism involving the impact of combinations of FSHR and LHCGR variants on receptor function. When analyzing a combination of the Asn680Ser of the FSHR and Asn312Ser of the LHCGR *in vitro*, granulosa cells from the group of women who were homozygous for Asparagine in both polymorphisms displayed a lower cAMP activity. Further, women homozygous for LHCGR Asn312 also required lower doses of exogenous FSH for an adequate response, and higher sensitivity of the receptor was attributed to the Asparagine (20, 21). Several studies have also indicated that high LH levels are associated with increased miscarriage rates and lower chances of pregnancy (31, 32), and it seems likely that a more sensitive LHCGR would have the same effect.

So controlled LH supplementation, based on the polymorphisms in the study Group-II, has resulted in significant improvement in clinical pregnancy rate (P-value: 0.049) and a trend showing improvement in live birth rates (P-value: 0.082). These observations are in line with the above-referred findings that LHCGR with Asn312 (A/A) variation has higher hormone sensitivity and any excess of LH concentrations has deleterious effects on the success rates.

Therefore, LHCGR polymorphism plays an important role in regulating the LH response and gains more significance as a pharmacogenomic-tool in ovarian stimulation. This directly impacts the choice of LH supplementation protocol, especially at a time when the need and dosage of LH supplementation in IVF cycles are still open to debate. The concept of categorizing a prior hypo-responder as a patient needing LH supplementation is not optimal as it involves wastage of an IVF cycle resulting in financial loss and emotional turmoil for the couple along with age-related negative effects during the subsequent cycles. The hypo-response could be related to specific genetic profiles of both the FSH and LH systems (33–35).

Another significant finding is that withholding LH in the A/A group had a beneficial effect. In line with our hypothesis on the role of LHCGR polymorphism in LH supplementation published in 2018, the data showed that there is a significant increase in success rate, even when the same patients received LH supplementation based on genetic data. The increase in success rate is observed despite the advanced age of patients by the time they have undergone the second IVF cycle. As there is no variation in the FSH dosage in these groups, results add to the existing evidence with regards to LHCGR gene polymorphism alone. Hence, the differences in observed pregnancy rates were attributed to the controlled stimulation protocols prior to IVF treatment.

In conclusion, the current study provides strong evidence in support of supplementation of LH to women undergoing IVF, based on their SNP profile (rs2293275) of LHCGR. This stimulation regimen provides the optimum levels of LH supplementation needed to improve the pregnancy and live birth rates.

## Data Availability Statement

The raw data supporting the conclusions of this article will be made available by the authors, without undue reservation.

## Ethics Statement

The studies involving human participants were reviewed and approved by Krishna IVF Clinic. The patients/participants provided their written informed consent to participate in this study.

## Author Contributions

1. RG: He is the project director and involved in the design of the study, dealing with the patients, counseling, and manuscript preparation. He played active role in the research, analysis and its outcome. 2. RC: He is involved in genetic and statistical analysis along with manuscript preparation 3. MK: He is the chief embryologist and played an active role in the IVF procedures involved in the study, and in the preparation of the manuscript. 4. KP: She is mainly involved in the follicle development monitoring and stimulation process. 5. KB: She is closely involved in monitoring IVF positive patients and fetal growth. 6. PM: She is involved in patient counselling and management of patient clinical data. 7. ST: He is in CASA and ICSI procedures. 8. AL: She played a crucial role in the review of the manuscript and inputs on recent advancements in the field. 9. SPRM: He is the key person in the preparation of the manuscript, monitoring the progress of the project and provided necessary suggestions on key issues in the project. All authors contributed to the article and approved the submitted version. 

## Funding

The authors declare that this study received funding from Krishnaivf clinic. The funder was not involved in the study design, collection, analysis, interpretation of data, the writing of this article or the decision to submit it for publication.

## Conflict of Interest

The authors declare that the research was conducted in the absence of any commercial or financial relationships that could be construed as a potential conflict of interest.
